# Silencing by the HUSH Epigenetic Transcriptional Repressor Complex

**DOI:** 10.1146/annurev-biochem-020425-045352

**Published:** 2025-06-01

**Authors:** Paul J. Lehner

**Affiliations:** Cambridge Institute of Therapeutic Immunology & Infectious Disease (CITIID), Jeffrey Cheah Biomedical Centre, Department of Medicine, University of Cambridge, Cambridge, United Kingdom

**Keywords:** HUSH complex, retroelements, retrotransposition, epigenetic silencing, intronless DNA, self–nonself discrimination

## Abstract

Retroviral and retrotransposon invasion pose a constant threat to genome integrity and have driven the evolution of host defense pathways able to counter these attacks. The human silencing hub (HUSH complex) is an epigenetic transcriptional repressor complex that recognizes and silences newly integrated retroelements through the establishment of ectopic heterochromatin and chromatin compaction. HUSH provides a genome-wide immunosurveillance system whose challenging task is to detect and silence any newly integrated retroelements, and it thus plays a key role in host defense. To distinguish self from nonself genomic DNA, HUSH recognizes long single-exon (intronless) DNA, the essential hallmark of reverse transcription. Retroelements, being RNA derived, lack classical, noncoding cellular introns, so a long, intronless sequence of DNA is the abnormal molecular pattern that allows HUSH to distinguish invading retroelements from intron-containing host genes. As a newly identified component of the innate immune system, HUSH protects the genome from the reverse flow of genetic information from RNA to DNA, revealing an unanticipated mechanism of postintegration genome immunity.

## Introduction

All organisms need to defend their genome from invasion by foreign nucleic acids, as they threaten their very integrity. To this end, higher eukaryotic cells have evolved a complex array of defenses, including specialized nucleic acid receptors that allow them to recognize and discriminate self from nonself DNA and RNA and therefore provide protection from nucleic acid invasion. None of these defenses are infallible, and many intracellular parasites have evolved strategies to overcome host defense pathways and promote their replication. Perhaps of greatest danger to the host is invasion of the genome itself, with the potential for genome damage and, if the germline is invaded, vertical transmission to subsequent generations.

One of the more remarkable findings to emerge from advances in genome sequencing is the sheer abundance of transposable elements (1–3). In the human genome, more than 40% of genomic DNA is derived from invading retroelements, while less than 2% is protein coding. From an evolutionary perspective, and despite its inherent dangers, invasion of the genome is therefore not an infrequent event. Somewhat to the contrary, genome invasion is clearly tolerated or inevitable, depending on one’s viewpoint. Either way, genome invasion provides an effective means by which complex organisms acquire new genetic material, and it can serve as a genetic reservoir from which new genes and regulatory elements arise (4, 5). The nature of the arms race between invading pathogens and their potential host suggests the potential existence of postintegration genome defense pathways, able to sense new integrations and mount a protective host response that involves silencing of the ectopic transgene (6).

DNA silencing or repression of invading nucleic acids provides an effective means to limit any immediate damage arising from genome invasion while at the same time providing the host genome with new genetic material (6). For multicellular organisms, retrotransposition, in which an RNA retroelement is reverse transcribed to a complementary DNA (cDNA) copy that then integrates into the genome, provides the most frequent route by which cells acquire new genetic material. Complex silencing pathways have evolved to control these transposable elements and are predominantly DNA or RNA mediated (7–10). The DNA repressor pathways are conceptually easier to understand, as illustrated by the large family of KRAB–zinc finger (ZNF) transcriptional repressors, which are characterized by an N-terminal KRAB domain and a specificity-determining C-terminal array of zinc fingers (11, 12). Each zinc finger interacts with a nucleotide triplet in the DNA target, thus conferring sequence specificity. The KRAB domain binds TRIM28, which in turn recruits the SETDB1 histone methyltransferase, which deposits repressive trimethylated histone 3 lysine 9 (H3K9me3) heterochromatin and establishes a transcriptionally silent state (13–15). Nuclear small RNA–silencing pathways are widely conserved across eukaryotes and provide an alternative to DNA-binding factors as specificity determinants in heterochromatin assembly (8, 16). RNA-silencing pathways can detect and respond to nucleic acid invasion and regulate genome stability through the formation of repressive heterochromatin. RNA-silencing pathways most commonly involve the biogenesis of a small RNA and its loading onto an Argonaute/Piwi family member, from where the bound RNA acts as the specificity factor that guides effectors to the transposable elements and induces gene silencing.

A shared feature of these different silencing pathways is that they predominantly silence elements that they have been trained to recognize, i.e., elements to which they have been previously exposed, thus representing a form of learned or adaptive immunity (17). While clearly effective, such silencing pathways are less able to recognize and repress novel transgenes, i.e., newly integrated foreign nucleic acids that they have never previously encountered and for which they therefore have no knowledge or memory. In vertebrates, this is the function of the HUSH complex (18), a heterotrimeric epigenetic transcriptional repressor complex whose challenging task is to repress retroelements to which the organism has never been previously exposed and has not therefore had time to evolve a more tailored defense strategy. HUSH first appears in cartilaginous fish, but its origins can be traced back to the well-characterized RNA-induced transcriptional silencing complex (RITS complex) of fission yeast (*Schizosaccharomyces pombe*) (19, 20). HUSH’s unique function is its ability to recognize and rapidly shut down transcriptionally active retroelements that have entered the nucleus, in particular those that have invaded the genome (17). At first glance it is not immediately obvious what features allow a silencing complex to distinguish self from nonself DNA once integration into the genome has occurred. Here, I describe the discovery of the HUSH complex, the unique features of newly integrated transgenes that HUSH recognizes and responds to, and our current understanding of how HUSH works.

## Discovery of the HUSH Complex

**Figure 1:**
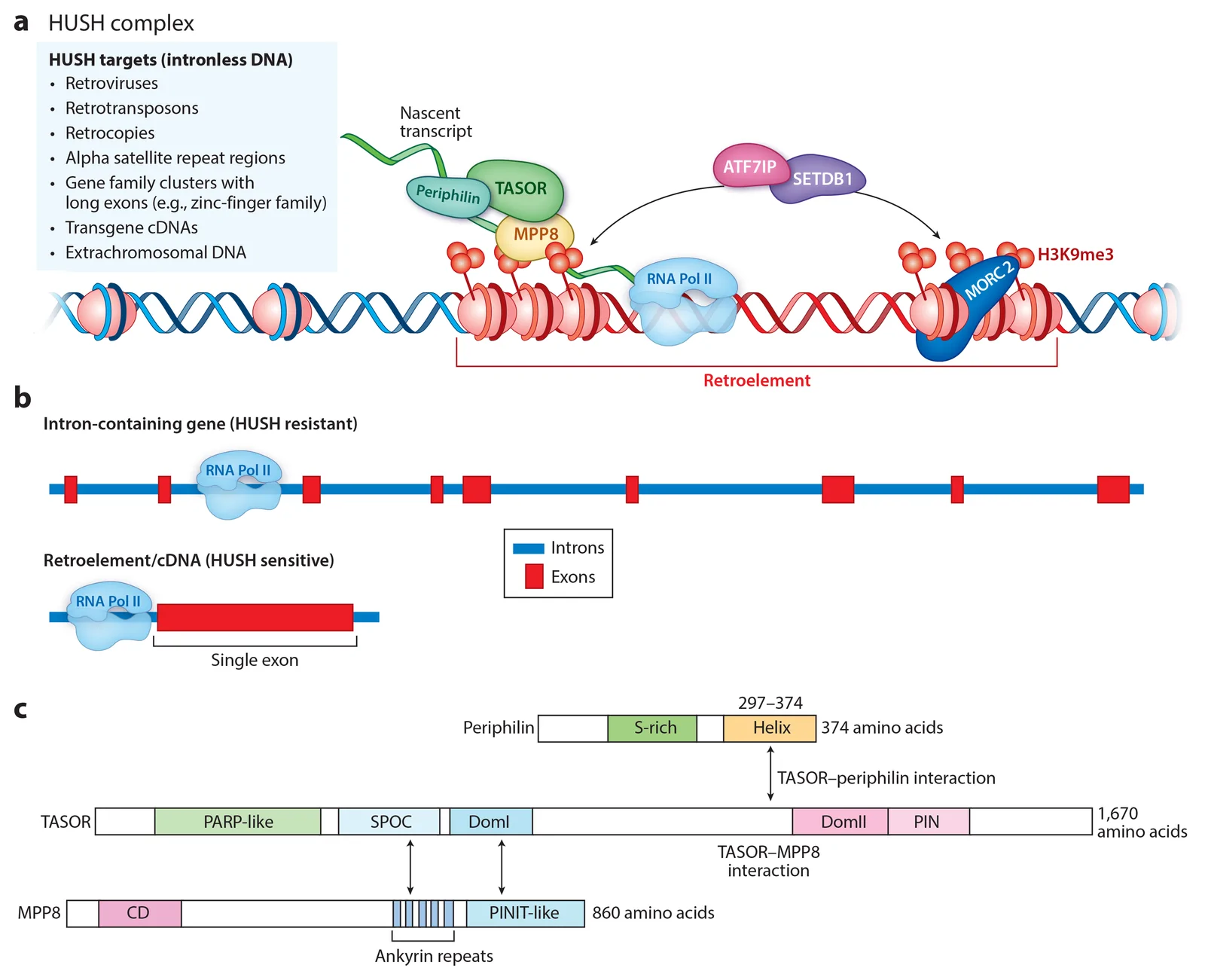
The HUSH epigenetic repressor complex and interaction sites. (*a*) Schematic of HUSH-mediated repression: Periphilin binding to nascent transcript both initiates HUSH recruitment and provides specificity for the locus to be silenced. MPP8 can then recruit SETDB1 and ATF7IP, which leads to H3K9me3 deposition, which reenforces MPP8 binding due to its CD, thus maintaining HUSH at the locus. Recruitment of the ATP-dependent MORC2 remodeler promotes chromatin compaction of the locus. (*b*) HUSH differentiates intron-containing genes (self) from single-exon intronless retroelements and cDNAs (nonself). (*c*) Schematic of the HUSH complex (TASOR, MPP8, and periphilin) showing domain organization and interaction sites. Abbreviations: CD, chromodomain; H3K9me3, trimethylated histone 3 lysine 9; HUSH, human silencing hub; MORC2, microrchidia family CW-type zinc finger 2; MPP8, M-phase phosphoprotein 8; RNA Pol II, RNA polymerase II; SETDB1, SET domain bifurcated histone lysine methyltransferase 1; TASOR, transgene activator suppressor. Figure adapted from Seczynska & Lehner (17) (CC BY 4.0).

The advent of whole-genome screening technologies in human cells allowed the application of unbiased genetic approaches to probe the genetic basis of retroelement silencing. Taking advantage of forward genetic screens in haploid human cells (21), the Lehner lab (18) used an integrating lentiviral reporter to identify genes required for the silencing of integrated retroviruses. This approach led to the discovery of HUSH as a new epigenetic transcriptional repressor complex and regulator of position effect variegation. They showed that the core HUSH complex is composed of the three nuclear proteins—TASOR (transgene activator suppressor), MPP8 (M-phase phosphoprotein 8), and periphilin—which recruit the two HUSH effector proteins, SETDB1 (SET domain bifurcated histone lysine methyltransferase 1) and MORC2 (microrchidia family CW-type zinc finger 2) (Figure 1a). SETDB1 deposits H3K9me3 (18), the canonical mark of repressive heterochromatin, while MORC2 is an ATP-dependent chromatin remodeler that initiates chromatin compaction (22); together, these two effectors help silence newly integrated retroviruses. The other key players in the retroelement family are the retrotransposons, which differ from the retroviruses, as they remain resident in the host cell. Like retroviruses, retrotransposons mobilize via an RNA intermediate that is reverse transcribed to cDNA, and this copy reintegrates back into the same genome, without ever exiting the host cell. Both retroviruses and retrotransposons encode their own reverse transcriptase enzyme, which allows them to reverse transcribe their RNA back to cDNA prior to integration into their host genome.

LINE-1 (L1) retrotransposons remain active within the human genome and represent some of the most successful host parasites, making up approximately 17% of the human genome (23, 24). Taking a genome-wide, CRISPR-based genetic screening approach, the Wysocka group (25) identified the same previously identified HUSH components as playing a key role in silencing an inducible L1 reporter, as well as endogenous L1s. Pursuing a more targeted approach, the Rowe group (26) also identified L1s as silencing targets of HUSH. Therefore, the HUSH complex plays a central role in recognizing and silencing newly integrated retroelements, irrespective of whether they originated outside the cell as a retrovirus or from within the cell as an endogenous retrotransposon (L1s).

HUSH’s ability to recognize and silence newly integrated L1 elements, regardless of where they integrate in the host genome, implied that silencing could be position independent. Initially this seemed at odds with HUSH’s original described function of silencing viruses that integrate in the vicinity of H3K9me3 heterochromatin, which is position dependent. The recognition and deposition of H3K9me3 on a transgene that has integrated into a locus already rich in surrounding H3K9me3 heterochromatin would seem mechanistically less challenging than the task of silencing a transgene that has integrated in actively transcribed euchromatin in the absence of any surrounding heterochromatin (27, 28). Yet L1 integrations do indeed occur in the introns of actively transcribed genes and are silenced in a HUSH-dependent manner, suggesting there is a position independent, sequence-specific component to HUSH-dependent silencing of L1 elements.

Further confusion arose from the unanticipated finding that HUSH also silences cDNA transgenes when expressed on reporters that have integrated into the host genome (29). This silencing was also position independent and included cDNA transgene sequences derived from the cell’s own genes (self-cDNA), as well as foreign cDNA sequences to which the cell had never previously been exposed. It was not immediately obvious how to reconcile these findings, in particular the wide variety of seemingly disparate HUSH substrates. While the cell’s ability and even requirement to recognize retroelements as invasive, foreign DNA seemed intuitive, it remained unclear why and how such a broad range of DNA transgenes (L1 elements, retroviruses, and self-cDNAs) could all be recognized as natural HUSH substrates.

## Self Versus Nonself Discrimination in the Genome: A Central Role for Introns

### HUSH-Repressed Loci Are Transcriptionally Active

The central issue therefore became, how does HUSH distinguish nonself from self-DNA once it has integrated into the human genome, i.e., what unique features allow self-versus-nonself discrimination following genome integration? Despite the inherent degeneracy of the genetic code, synonymous codon usage in the genome is not random, and all DNA does not look the same (30, 31). Genomes of different organisms show distinct codon biases, with genomic sequences maintained under tight evolutionary constraints, reflecting both natural selection and neutral genetic drift. Selection on synonymous sites, leading to nucleotide bias, may not only affect gene expression but also provide features that allow discrimination between self and nonself nucleic acid (31).

Given the general silencing capabilities of HUSH, with its ability to repress a wide variety of substrates, it seemed most likely that HUSH was recognizing a general feature of nonself nucleic acid, rather than a sequence-specific motif within the invading DNA. Further analysis of HUSH targets identified the following features as central to HUSH-mediated repression: (*a*) a transgene length >1 kb, (*b*) a requirement for active transcription, and (*c*) an adenine (A)-rich nucleotide sequence in the sense strand, i.e., the strand undergoing RNA Pol II transcription (29).

An absolute requirement for HUSH-sensitive loci to be transcriptionally active arose from the observations that (*a*) only young, full-length, and therefore transcriptionally active L1s were HUSH sensitive (25, 26); (*b*) increasing target transcription enhanced HUSH binding (25); and *(c)* removal of a transgene promoter, with the resulting loss of transcription, led to the loss of H3K9me3 occupancy over a previously HUSH-sensitive locus (29). While it initially seemed somewhat counterintuitive that a locus to be silenced must at the same time be transcriptionally active, ongoing transcriptional activity is a feature of other RNA-based silencing systems and has the advantage of allowing HUSH-mediated silencing to be fine-tuned to the transcriptional activity of the locus. HUSH therefore acts as an epigenetic, transcription-responsive rheostat: Loci with the potential to be transcriptionally active are monitored, and if transcribed, they are silenced by HUSH. In contrast, transcriptionally inactive loci are unlikely to cause problems and are ignored, requiring neither HUSH occupancy nor repressive histone marks. HUSH therefore provides a genome-wide surveillance system to detect and silence invasive transgenes. However, none of the features described above help explain the key event, the recognition of nonself DNA.

### Intronless DNA Provides the Molecular Pattern Recognized by HUSH

**Figure 2:**
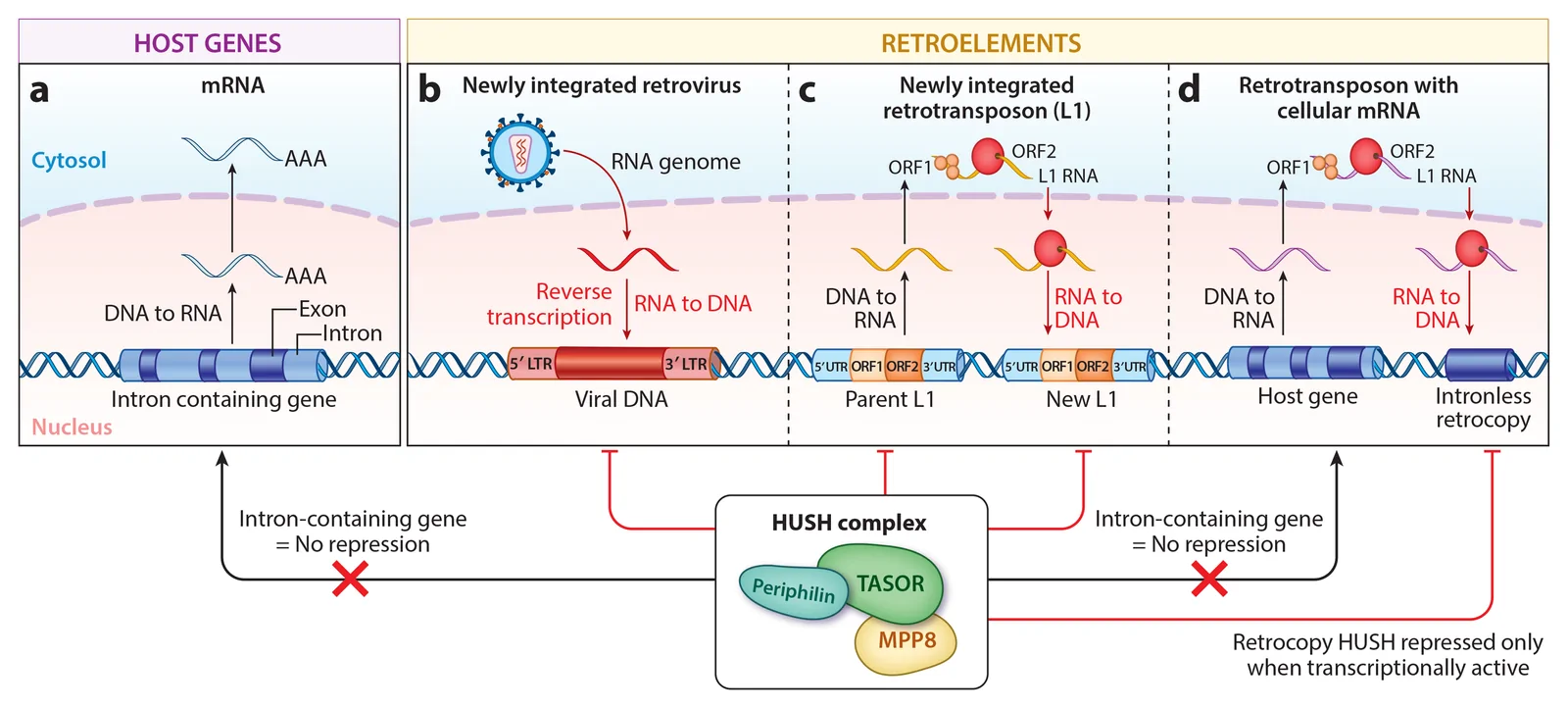
The HUSH complex targets large single-exon (intronless) genes. (*a*) Intron-containing mammalian genes are not targeted by HUSH. (*b*–*d*) HUSH silences long single-exon (intronless) genes, most commonly retroelements that have been reverse transcribed from RNA to DNA and then integrated into the genome and include (*b*) integrated retrovirus, (*c*) integrated retrotransposons (L1 elements), and *(d)* cellular retrocopies. Abbreviations: HUSH, human silencing hub; L1, LINE-1; LTR, long terminal repeat; MPP8, M-phase phosphoprotein 8; mRNA, messenger RNA; ORF, open reading frame; TASOR, transgene activator suppressor; UTR, untranslated region. Figure adapted from Seczynska & Lehner (17) (CC BY 4.0).

The remaining key feature of HUSH silencing is the presence of long, single-exon (intronless) DNA. This is of paramount importance, as genes within most complex genomes are intron rich, with introns comprising 80–90% of a mammalian gene (32). Genes are organized with a characteristic exon–intron structure such that the average exon (150 bp) is 40 times smaller than the average intron (6,000 bp), i.e., exons exist as small islands within a sea of introns that are removed during transcription (33). The absence of introns is therefore unusual and is a hallmark of retroelements, which, being RNA derived, have had any introns spliced out. Long, intronless DNA therefore provides the unique feature, the pathogen-associated molecular pattern (PAMP), that allows HUSH to distinguish self-DNA (intron containing) from recent retroelement-derived foreign DNA (intronless) (Figures 1b and 2). Introns therefore provide protection from HUSH-mediated repression, and importantly, insertion of an intron into a single-exon reporter converts a locus from being HUSH sensitive to HUSH resistant, with the concomitant loss of H3K9me3 (29). Somewhat surprisingly, this role for introns in providing protection against HUSH silencing does not appear to be related either to splicing or to recruitment of the splicing machinery (29). Indeed, reporters with splice sites mutated so that the intron is retained, but not spliced out, remain HUSH insensitive. In contrast, reporters that efficiently splice out a stuffer sequence, a non-intron derived DNA sequence, remain HUSH sensitive, despite effectively recruiting the splicing machinery (29).

Understanding the features of introns that provide protection again HUSH-mediated silencing remains an important and unanswered question. Since intron-mediated protection from HUSH is not related to splicing, it must relate to an inherent property of the intron itself. A large number of RNA-binding proteins (RBPs) are cotranscriptionally recruited to introns in nascent transcripts as part of the RNA processing machinery (34–37) and are required for both splicing and RNA nuclear export (38). These RBPs could compete, directly or indirectly, with periphilin for binding to nascent transcripts, accelerating their nuclear export and providing these transcripts with an escape route from chromatin and HUSH. While introns are characteristically adenine (A) and thymine (T) rich and exons are guanine (G) and cytosine (C) rich, there is extreme DNA strand asymmetry within introns, toward T and away from A (38, 39), which may help confer intron identity in nascent RNA. Importantly, T-rich nucleotides protect against HUSH (29), while A-rich nucleotide sequences are a key feature conferring sensitivity to HUSH silencing (29), as seen in retrotransposons such as L1s and HIV-1 lentiviruses (40–42). Whether T-rich introns help promote RNA export through RBP recruitment or exert a more direct effect on RNA Pol II elongation and HUSH silencing remains unclear.

Differences in RNA Pol II elongation in introns versus exons may also provide clues as to the protective effect of introns. RNA Pol II elongation is sensitive to nucleotide composition and especially to GC richness, with elongation rates slow through GC-rich exons and accelerating through introns (43–47). The decreased nucleosome occupancy of introns also correlates with faster RNA Pol II elongation (45, 48–50). Slow elongation of RNA Pol II through abnormally long exons could therefore provide the trigger for periphilin to bind nascent RNA with subsequent HUSH recruitment, which would be countered by the faster elongation rates in introns and would help explain the accumulation of HUSH at sites of high RNA Pol II occupancy (51).

Introns therefore provide an effective means to discriminate self from nonself DNA following retroelement integration into the host genome. This feature is exploited by HUSH to provide an autonomous genome immunosurveillance system that distinguishes nonself from self DNA. In so doing, HUSH provides a mechanism to protect against, but not prevent, the reverse transcriptase–dependent reverse flow of genetic information by silencing RNA-derived retroelements in the genome.

### Genomic HUSH Loci Are Intronless

While the rules that determine whether a specific locus is likely to be HUSH sensitive were predominantly established using reporter constructs, the validity of these predictions was subsequently confirmed through genomic analysis of HUSH-sensitive loci. Chromatin immuno-precipitation sequencing (ChIP-seq) analysis of HUSH-sensitive loci, from both human and mouse cell lines, identified the key loci targeted by HUSH as L1 elements, specifically evolutionarily young, transcriptionally active L1 elements (LIPA1–L1PA5) (25, 26); endogenous retroviruses and alpha satellite repeat regions (18, 52–54); retrocopies and processed pseudogenes (29); gene family clusters, most notably the KRAB-ZNF and the protocadherin gene clusters (18, 52); endogenous genes containing unusually long single exons, e.g., exon 11 of *BRCA2* (3.4 kb) and exon 3 of *AHNAK* (17 kb) (20); and cDNAs of expressed transgenes (29). The shared features of these loci are that either they are intronless (L1s, retrocopies, retroviruses, and transgene cDNAs) or they contain one unusually long exon, e.g., intron-containing KRAB-ZNF genes have a long 3^′^ exon (average length >4 kb) (55, 56) over which H3K9me3 is deposited, while the protocadherin genes have an unusually long first exon (2–4 kb) at their 5^′^ end (57). In the case of the intron-containing BRCA2 gene, HUSH-dependent H3K9me3 is deposited only over a single exon, the 3.4-kb exon 11 (20). Genome surveillance by HUSH therefore ensures that HUSH-dependent H3K9me3 is deposited over those loci where the normal exon–intron architecture is disrupted and the loci have acquired molecular features (long exons) that resemble retroelement insertions.

Genomic analyses in mouse embryonic stem cells (mESCs) identified an independent set of loci that, despite being bound by HUSH components, fail to effectively recruit SETDB1, are devoid of H3K9me3, and are not transcriptionally repressed by HUSH (51). In mESCs, these H3K9me3-negative loci are more abundant than H3K9me3-positive HUSH loci, with targets including single-exon histone genes, as well as tRNA genes, the majority of which are also intronless, and A-rich tRNA-derived repeats. As these loci are not transcriptionally repressed by HUSH, their functional significance remains unclear, but one of these H3K9me3-negative HUSH occupied sites could be triggered to full H3K9me3-positive HUSH silencing following the deletion of an intervening noncoding gene desert (51). Given the dynamic nature of HUSH surveillance, these sites may reflect different thresholds required for HUSH silencing. Whether H3K9me3-negative loci represent an intermediate stage in which HUSH is triggered to occupy a locus without reaching the threshold required for silencing remains to be determined. The presence of these H3K9me3-negative sites emphasizes how different loci are under continual HUSH surveillance. HUSH also plays an essential role in suppressing L1 expression in S phase. By interacting with the leading-strand DNA polymerase Pol ε, HUSH promotes asymmetric H3K9me3 distribution at head-on L1s to suppress their expression in S phase (58).

## The Molecular Basis of HUSH Silencing

### The Core HUSH Assembly Complex

TASOR, MPP8, and periphilin form the core components of the HUSH complex and are essential for HUSH-mediated silencing (Figure 1a). All three proteins had been implicated in epigenetic silencing prior to the identification of HUSH. MPP8 was originally described as a methyl-H3K9-binding protein (59–61), while periphilin was suggested to function as a transcriptional corepressor through an interaction with the Sin3–histone deacetylase 1 (HDAC1) corepressor complex (62, 63). The murine ortholog of TASOR (*D14Abb1e*) was identified in an in vivo chemical mutagenesis screen for modifiers of green fluorescent protein (GFP) transgene expression (64) and in an HIV-1 screen to identify novel cellular restriction factors of HIV-1 (65).

MPP8’s N-terminal chromodomain (CD) binds SETDB1 and represses E-cadherin expression (60) via the HDAC SIRT1 protein (66), while its C-terminal domain (CTD) consists of five ankyrin repeats followed by a domain with structural homology to the PINIT domains of Siz/PIAS-family SUMO E3 ligases (20, 67). Its CD binds H3K9-like sequences in ATF7IP, the SETDB1 nuclear chaperone (68), as well as H3K9-like peptides from a broad set of H3K9 methyl-transferases (67). This ability to both read, via CD binding to H3K9me3, and write, via recruitment of SETDB1, allows MPP8 to regulate H3K9me3 deposition. MPP8’s NTD also binds DNA methyltransferase 3A (DNMT3A) (60, 69) providing a potential link between histone (H3K9me3) and DNA methylation. Periphilin is a highly insoluble 55-kD nuclear protein with a disordered, self-aggregating NTD that is essential for HUSH activity (70) and has multiple isoforms. TASOR provides the central assembly platform of HUSH (Figure 1c), connecting MPP8 and periphilin, and is the largest (1,670 amino acids) of the three HUSH components. TASOR has three structured regions essential for HUSH function: an N-terminal, noncatalytic PARP-like domain; a SPOC domain; and a DomI domain (20, 71). The PARP-like, SPOC, and DomI domains are connected to the C-terminal DomII and Pin domains by a long unstructured central linker.

Biochemical analysis and AlphaFold models have helped define a minimal HUSH assembly unit in which an extensive TASOR–MPP8 interaction interface forms between the TASOR SPOC and DomI domains and the MPP8 CTD through a region that overlaps the start of MPP8’s ankyrin repeats (residues 500–560). TASOR (residues 1,000–1,085) also interacts with the C-terminal coiled-coil domain of a periphilin homodimer (20, 67, 70) (Figure 1c). This core HUSH complex is responsible for the identification of loci for silencing and the recruitment of effectors to mediate this silencing. While a structural understanding of the complete HUSH complex is not yet available, structural and AlphaFold models of the folded domains of HUSH required for repression show that MPP8 and TASOR form a heterodimer with two molecules of periphilin binding a single TASOR molecule in the core periphilin–TASOR complex, giving the total HUSH complex a stoichiometry of 1:1:2 MPP8:TASOR:periphilin (67).

Searching for proteins with a similar domain organization to TASOR revealed a striking homology and functional similarity with components of the RITS complex, the well-characterized yeast repressor complex of *S. pombe* (19). Like TASOR, the *S. pombe* Chp1 protein has SPOC, DomII, and PIN domains (20, 71), which together with its Tas3 binding partner, form the core structure of the RITS complex. RITS targets repeat regions in centromeres and telomeres and, like HUSH, mediates the deposition of repressive H3K9me3 over repeat elements in response to transcription (19, 72). Chp1 has an H3K9me3-binding CD, which is not found in TASOR but is present in MPP8. Furthermore, Tas3 resembles periphilin in its ability to self-associate through C-terminal helical repeats and to spread H3K9me3-mediated heterochromatin (70, 73).

Of the three core HUSH components, only MPP8 shows genome occupancy in the absence of other HUSH components, with long terminal repeats (LTRs) frequently targeted by MPP8 together with components of the TRIM28 repressor complex (26, 51).

### RNA Binding and the Role of Periphilin

A central role for RNA binding as an early recognition event in HUSH-dependent silencing was suggested by (*a*) the requirement for transcription and recognition of nascent transcripts for HUSH silencing; (*b*) similarities between HUSH and the RITS silencing complex; (*c*) the finding that silencing of identical DNA sequences was directional, i.e., orientation dependent, relative to transcription by RNA Pol II; and (*d*) the association of TASOR with components of RNA Pol II and the RNA-processing machinery (20, 51, 71). Although none of the three core HUSH components contain a well-defined RNA-binding domain, each HUSH component is reported to have some in vivo RNA-binding capability (20, 29, 51, 70, 71, 74, 75), though how much of this binding could be ascribed to an individual protein as opposed to the core HUSH complex was unclear. As full-length HUSH complex proteins are difficult to express in vitro, direct assessment of RNA-binding capacity has not yet been possible.

It was therefore essential to establish which HUSH component(s) bind RNA, particularly functionally relevant RNAs produced from HUSH target loci, and whether this RNA binding is a prerequisite for HUSH recruitment to chromatin and subsequent silencing; i.e., is HUSH binding to chromatin RNA directed, or is RNA binding by HUSH just a consequence of its proximity to ongoing nascent transcription?

RNA immunoprecipitation sequencing (RIP-seq) data showed that periphilin binds transcripts from HUSH target loci, making it the prime candidate required for HUSH chromatin localization through the stabilization of HUSH at its target loci (29). Further clarification of which HUSH components bind RNA came from an unbiased approach using RNA interactome capture (76), which identified periphilin as the sole HUSH component able to bind RNA alone, in the absence of other HUSH complex members (77). Periphilin is highly insoluble, with a disordered, self-aggregating N-terminal domain (NTD) that is essential for HUSH activity (70). This NTD is rich in arginine and tyrosine residues, and its functional activity can be partially complemented by low-complexity regions from other RBPs (70). RNA interactome capture alongside functional analysis showed that periphilin’s N-terminal 127 amino acids are required both for RNA binding and for HUSH activity, as deletion of these 127 amino acids leads to a loss of both these activities (77). Importantly, the RNA-binding requirement of periphilin can be effectively bypassed by directly recruiting periphilin to a nascent transcript. This approach takes advantage of the interaction between boxB RNA stem loops (inserted into a HUSH-resistant reporter construct) and lambda N peptides fused to the protein of interest (78), in this case individual components of the HUSH complex. Under these conditions, a periphilin–lambda N fusion protein was the only HUSH component capable of silencing an otherwise HUSH-resistant boxB-engineered reporter (77). Importantly, tethering periphilin–lambda N to the boxB stem loops on the reporter transcript allowed the first 291 residues of the 374-amino-acid periphilin to be deleted while retaining its silencing capability. A minimal 83-residue CTD, which includes the region where periphilin interacts with TASOR, is therefore required for HUSH-dependent silencing (77).

Therefore, periphilin binds RNA, and this binding is essential for HUSH activity. Directly recruiting periphilin to the nascent transcript of the locus to be silenced bypasses the RNA binding requirement. These data, together with genome-wide periphilin RIP-seq analysis showing that transcripts bound by periphilin are known HUSH targets (full-length L1s, KRAB-ZNFs, and HUSH-sensitive loci) (29), as well as transcripts derived from intronless genomic loci (29), strongly suggest that periphilin-bound RNA specifies the loci for HUSH repression, most likely by directing and stabilizing HUSH at these target loci.

The current evidence therefore suggests that periphilin binding to nascent RNA specifies the loci to be silenced. HUSH binds to key components of the transcriptional machinery, including RNA Pol II (20, 71), as well as components of the CPSF complex (FIP1L1) and the WDR82 protein, which directly associates with the RNA Pol II CTD to regulate transcription termination (51). HUSH may therefore track RNA Pol II, with binding to the WDR82 and CPSF transcriptional termination components regulating its genome occupancy (51). Indeed, in mESCs, WDR82 deletion reduces HUSH locus occupancy, leading to transcriptional readthrough, suggesting that HUSH targeting is coupled to the transcription termination machinery. In wild-type cells with intact WDR82, transcriptional termination may result in the production of prematurely terminated nonpolyadenylated RNAs, which are potential substrates for degradation by the nuclear exosome targeting (NEXT) complex, part of the RNA-degradation machinery (see the section titled HUSH as a Central Hub to Recruit Effectors to Nascent RNA).

### HUSH Recruits Effectors to Silence Sensitive Loci

Following engagement with its target loci, HUSH recruits its key effector proteins: SETDB1 and MORC2 (18, 22). SETDB1 deposits repressive H3K9me3 heterochromatin (79, 80) while MORC2 compacts the repressed locus (81), so that together these two effectors silence newly integrated retroelements. The CD of MPP8 binds both H3K9me3 and ATF7IP, the SETDB1 nuclear chaperone (68, 82, 83). In the simplest read–write scenario, recognition of H3K9me3 by MPP8’s CD would then recruit the ATF7IP–SETDB1 complex with further H3K9me3 deposition. However, the situation is clearly more complex and likely to be context dependent. A simple read–write scenario does not account for the absolute requirement for TASOR and periphilin in HUSH-mediated silencing. It also does not account for the enrichment of HUSH target genes to actively transcribed chromatin (25, 26, 84), where HUSH must be able to silence ectopic loci that have inserted into actively transcribed genes in the absence of neighboring H3K9me3 heterochromatin. Furthermore, while the MPP8 CD is required to establish silencing, neither the CD nor ATF7IP methylation appear to be absolutely required for maintenance of silencing over some loci (18, 53). MPP8 CD mutants are able to maintain silencing over GFP reporters and endogenous L1 elements, and an increasing number of HUSH-sensitive loci show no absolute requirement for H3K9me3 (53), with repression maintained in the absence of H3K9me3. Therefore, while H3K9me3 seems likely to contribute to silencing, other effector molecules, such as MORC2, are able to maintain the repressed state, suggesting there might be additional functions for H3K9me3 in the context of HUSH silencing. The most obvious explanation would be to protect HUSH loci from unwanted recombination events, which commonly arise following gene duplications due to sequence homology, as seen with both the rapidly emerging ZNF family cluster on chromosome 19 (56) and the protocadherin gene cluster on chromosome 5 (57), many members of which are key HUSH targets. As recently discussed (17), H3K9me3 might also facilitate HUSH–RNA binding by decreasing RNA Pol II elongation rates (44) and retaining nascent transcripts on chromatin.

An essential role for the MORC2 ATP-dependent chromatin remodeler in HUSH silencing emerged from an independent CRISPR screen (22), and MORC2 has subsequently been implicated in the silencing of most known HUSH loci (25, 53, 81). HUSH recruits MORC2 to target sites in heterochromatin, and MORC2 depletion phenocopies the loss of core HUSH components with chromatin decompaction, loss of H3K9me3 deposition, and transcriptional derepression (22). MORC2 chromatin occupancy is itself markedly reduced in the absence of individual HUSH components. MORC2 has an N-terminal GHKL-type ATPase module and a coiled-coil insertion (CC1), which form ATP-dependent dimers essential for HUSH activity (81). Biochemical and structural analyses of this N-terminal region show how monomers of MORC2 bind dsDNA with high affinity via the CC1 projection. MORC2 cycling between the monomeric and dimeric states brings two DNA duplexes together, promoting DNA loop formation and chromatin compaction (81). Naturally occurring mutations in the ATPase domain of MORC2 are associated with human neuropathies—most commonly Charcot–Marie–Tooth Disease (CMT), with gain of function CMT mutations causing hyperactivation of HUSH activity with enhanced repression of target loci in neuronal cells (22). Deletion of *Mphosph8* or *Morc2a* in the murine nervous system results in enlarged brain size, modified brain structure, and behavioral changes. In mouse brains and human cerebral organoids, MPP8 and MORC2 inhibit the repetitive protocadherin gene cluster through an H3K9me3-dependent mechanism (52). The purification of full-length human MORC2 uncovered a second DNA-binding homodimerization interface at its C-terminus. Dimerization of the NTD and CTD would then allow MORC2 to encircle DNA substrates like a clamp, with DNA being held within the lumen of the dimers, a unique mode of DNA binding that may limit DNA accessibility and therefore impede transcription (85).

### HUSH Links Histone Methylation with DNA Methylation

The combination of SETDB1-dependent H3K9me3 deposition and MORC2-dependent chromatin compaction is therefore likely to be sufficient to mediate HUSH-dependent silencing. Communication between histone methylation via the HUSH complex and DNA methylation was suggested from work in mESCs (26), based on the original reports that MPP8 recruits DNMT3A. The HUSH complex, via MPP8, could therefore provide a link between histone and DNA methylation (69), providing a molecular explanation for the co-occurrence of the two systems (86, 87). This connection would be of particular importance as DNA methylation is strongly correlated with transcriptional silencing of repeat regions (88, 89). The relationship between HUSH silencing and DNA methylation was explored in human neural progenitor cells (90). This somatic cell model of human brain development has the advantage of showing robust DNA methylation and tolerating deletion of DNMT1, the enzyme that maintains CpG methylation during cell division. In this cell type, the majority of L1s were silenced by DNA methylation, with only a limited number of demethylated L1 copies weakly transcribed and therefore restricted by MORC2/HUSH. However, loss of DNMT1 and the ensuing transcriptional activity sensitized the major HUSH loci (young L1 elements, alpha satellite repeat regions, and the protocadherin gene cluster) to MORC2/HUSH accumulation, while the combined depletion of DNMT1 and MORC2 led to a marked transcriptional increase of these elements (90). This would suggest a dual layer of epigenetic control, with a mechanistic hierarchy, in that the majority of L1 elements are silenced by DNA methylation with no longstanding requirement for HUSH. These loci are likely to attract HUSH activity if DNA methylation is lost, as occurs in the demethylation phases of epigenetic reprogramming during embryonic development. How widespread these effects are remains to be determined.

### HUSH as a Central Hub to Recruit Effectors to Nascent RNA

As well as recruiting effectors for histone and DNA methylation, HUSH has acquired additional functions and recruits components of the RNA-degradation machinery: the NEXT complex (91) and the CNOT1 RNA-degradation pathway (71). The NEXT complex guides the exosome-mediated degradation of short, nonpolyadenylated Pol II–derived RNAs, and one of its three components, the zinc finger protein ZCCHC8, is recruited to HUSH via MPP8 (92, 93). HUSH cooperates with the NEXT complex to promote the degradation of prematurely terminated RNAs arising from HUSH-repressed L1s and endogenous retrovirus elements (91, 93). HUSH, together with NEXT, can therefore limit the expression of retroelements through a combination of transcriptional silencing and posttranscriptional degradation. The biological importance of NEXT complex activity with HUSH is unclear, as the nonpolyadenylated substrates of NEXT are themselves too short to allow mobilization in *cis* (93). In a similar manner, HUSH cooperates with the CNOT1 RNA-degradation pathway to repress latent HIV-1 (71).

**Figure 3:**
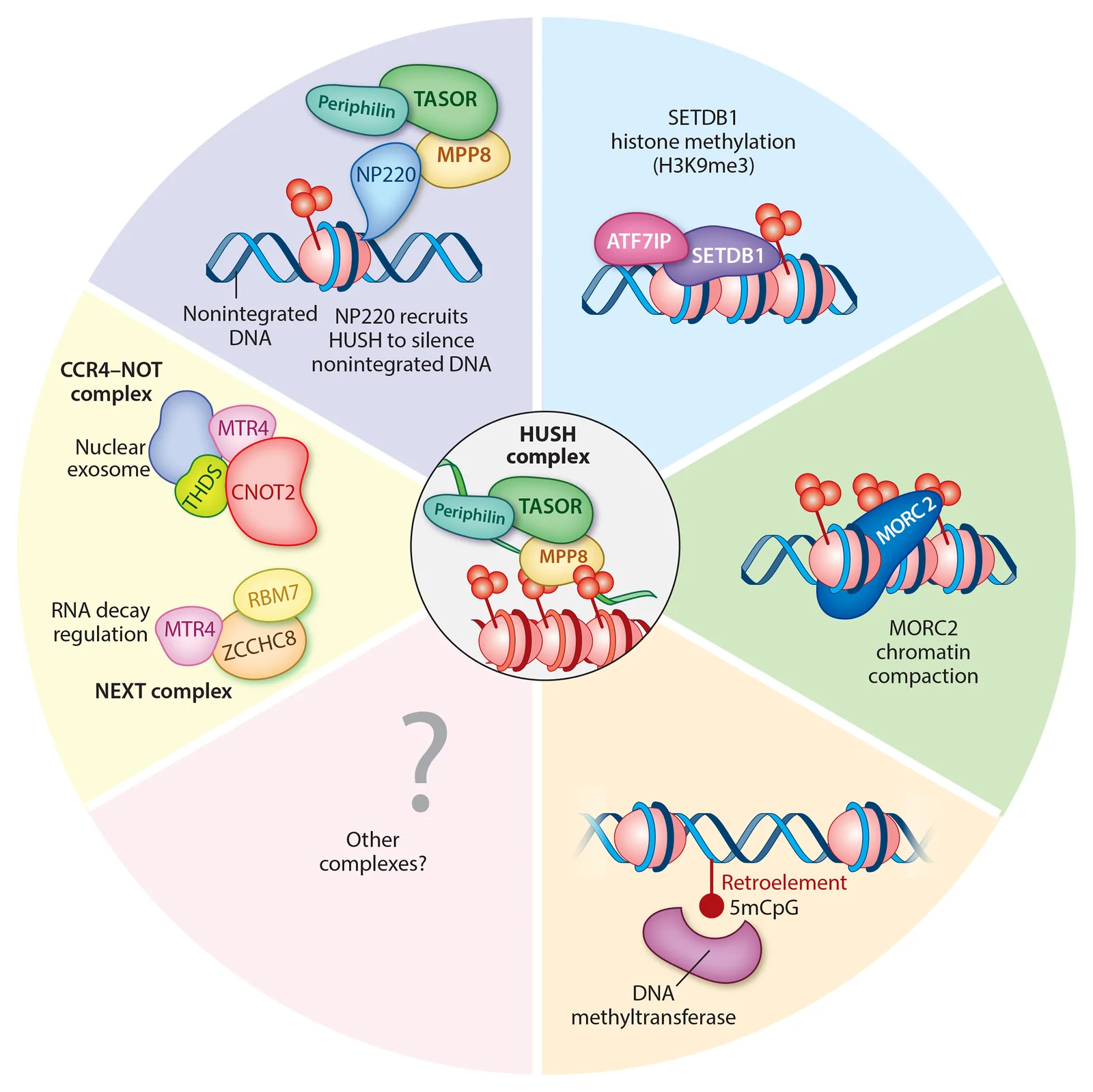
The HUSH complex acts as a hub to recruit effectors. Recognition of foreign, nonself DNA is the central function of the core HUSH complex. HUSH recruits several effector complexes including but not limited to SETDB1 and ATF7IP for deposition of repressive H3K9me3 chromatin; MORC2, leading to chromatin compaction; DNA methylation machinery for DNA methylation and silencing; and the NEXT complex and CCR4–NOT complex to promote degradation of short, nonpolyadenylated RNAs. Alternatively, NP220 binds to DNA and recruits HUSH via MPP8 to promote silencing of non-integrated DNA. Abbreviations: 5mCpG, 5-methylcytosine in CpG DNA; H3K9me3, trimethylated histone 3 lysine 9; HUSH, human silencing hub; MORC2, microrchidia family CW-type zinc finger 2; MPP8, M-phase phosphoprotein 8; NEXT, nuclear exosome targeting; SETDB1, SET domain bifurcated histone lysine methyltransferase 1.

The picture that emerges is that the HUSH complex acts as a central hub that takes advantage of its unique ability to recognize nonself/foreign transgenes and is then able to recruit different effector pathways, which together can limit retroelement expression (Figure 3). These involve the transcription-dependent silencing of newly integrated transgenes through SETDB1 histone methylation and MORC2-mediated chromatin compaction, DNA-mediated silencing through DNMT1 and recruitment of DNA methylation machinery, and cotranscriptional RNA degradation through the NEXT complex and recruitment of RNA-processing complexes, with alternative effector pathways also envisaged.

### A Model for HUSH Activity

HUSH provides a dynamic transcription-dependent immunosurveillance system, continuously monitoring the genome for the presence of retrotransposed foreign DNA. Our current model suggests this is achieved by HUSH tracking the transcriptional activity of RNA Pol II and associated components of the transcriptional processing machinery, including the WDR82 transcription termination factor. Aberrant transcription through unusually long, A-rich exons is sensed as abnormal, as foreign, initiating a signal that is detected by HUSH, that in turn triggers periphilin to bind the nascent transcript and direct HUSH in *cis* to the locus to be silenced. Self-aggregation of periphilin through its arginine-tyrosine-rich RNA-binding NTD then initiates the formation of large ribonucleoproteins and higher-order molecular condensates (70), enabling nucleation of the HUSH complex at the required site and recruitment of its effector molecules SETDB1 and ATF7IP to initiate formation of H3K9me3 heterochromatin. This is helped by MPP8’s CD binding either to neighboring H3K9me3 or via ATF7IP and SETDB1, leading to further H3K9me3 deposition. DNA accessibility and transcriptional silencing is further reinforced through the recruitment of MORC2, leading to DNA locus compaction. As long as the locus remains intact and retains transcriptional activity, HUSH occupancy, ectopic heterochromatin, DNA compaction, and silencing are maintained. Over time, different scenarios may ensue: If transcription decreases, HUSH binding and H3K9me3 are lost, as may occur if the locus decays and loses transcriptional activity. If its promoter becomes DNA methylated and loses all transcriptional activity, the locus remains silenced but loses HUSH binding. Alternatively, the transposable element may acquire new biological function(s), facilitated by the binding of DNA-sequence specific KRAB-ZNFs, which may both repress (94, 95) and coordinate the locus’s regulatory potential (96–99). This scenario can be seen with some L1s that are targeted by both KRAB-ZNFs and HUSH, potentially as part of an ongoing domestication process (26, 51). The prediction here is that, following a period of shared locus occupancy, the KRAB-ZNFs will eventually dominate, with the subsequent loss of HUSH binding.

While nascent RNA binding by periphilin specifies the loci to be silenced, how transcription through long A-rich exons is sensed as abnormal and then initiates RNA binding remains unclear, but it is unlikely to rely solely on periphilin. It would seem more plausible that RNA binding is stimulated by an upstream triggering event, relating to a change in RNA Pol II behavior and transcription dynamics. This change would need to be sensed by a HUSH component, most likely TASOR, triggering periphilin to bind the nascent transcript. For example, RNA Pol II elongation is sensitive to the local environment, transcribing faster through AT-rich introns and slower through GC-rich exons (43). Elongation rates therefore fluctuate as the polymerase transcribes through exons and introns, with even short exons eliciting slower rates of elongation (43). Elongation through long, densely packed nucleosome-rich exons is likely be particularly challenging without introns to allow RNA Pol II time for recovery and could trigger TASOR to initiate RNA binding by periphilin, potentially explaining why HUSH accumulates at sites of high RNA Pol II occupancy (51). In this sense, HUSH acts as a checkpoint and part of the transcriptional quality control machinery, marking an ectopic retroelement within an actively transcribed gene as a danger signal for RNA Pol II, yet one through which the polymerase must be able to transcribe to achieve productive elongation. HUSH occupancy may therefore provide a warning signal to the RNA Pol II transcriptional machinery.

## A Comparison of HUSH With Other RNA Silencing Systems

RNA, in particular small RNA molecules, has a well-established role in sequence-specific gene regulation. Given that HUSH-dependent silencing is RNA mediated, how does HUSH compare with other RNA-dependent silencing systems? It is instructive to compare HUSH with the well-characterized RITS complex of *S. pombe*, which also silences centromeric DNA repeat regions and shares structural and protein domain organization similarities with HUSH components. In the current model for RITS, small interfering RNAs (siRNAs) bind to their Argonaute-binding proteins, assemble into the RITS complex, and direct epigenetic chromatin modifications and heterochromatin formation at nucleotide complementary sites (8). RITS is targeted to chromatin through base pairing between siRNA and RNA Pol II–derived nascent transcripts of the repeat DNA sequence to be silenced, with this association stabilized by direct binding to methylated H3K9. Upon nascent transcript target recognition, RITS recruits the RNA-dependent RNA polymerase (RdRp) complex, which synthesizes dsRNA using the targeted transcript as template, producing additional substrate for siRNA processing by the Dcr1 ribonuclease. The resulting positive feedback loop reenforces the silencing signal. These two activities of RITS, siRNA base pairing to nascent RNA transcripts and Chp1-mediated binding to chromatin via methylated H3K9, are therefore able to initiate heterochromatin formation (8, 100–102).

Like RITS, and similar to other RNA-silencing systems, HUSH binding to nascent transcripts initiates ectopic heterochromatin formation. However, HUSH differs from RITS and other silencing systems, as there is no identified role for processing small RNAs or binding to Argonaute proteins. Importantly, RITS and other small RNA-silencing complexes use the RdRp enzyme to amplify the RNA signal, and while this enzyme is found in a variety of organisms, including viruses, fungi, plants, and worms (103, 104), it is absent from insects and vertebrates. Similarly, Piwi-interacting RNA (piRNA) silencing systems amplify their RNA signals through the ping-pong cycle (105, 106), but this is not a known feature of HUSH-dependent silencing. It is therefore intriguing that HUSH has no identified mechanism for reinforcing the RNA silencing signal, making it important to determine whether it has evolved an alternative mechanism of RNA amplification that may, for example, involve the self-aggregating properties of periphilin.

Another distinctive feature of HUSH is TASOR’s DUF3715/noncatalytic PARP domain, which is essential for HUSH activity but of unclear function. This domain is not identified in any RITS components and is likely to have evolved later, as it can be found in the TEX15 protein of piRNA silencing pathways. piRNAs identify active transposon loci in the germline by binding their nascent transcripts and tethering piRNA-bound MIWI2 to nascent RNA (106) with TEX15, a MIWI2-associated factor essential for silencing (107, 108). piRNA pathways, like HUSH, require nascent transcription for silencing and also recruit MORC proteins as effectors to enable silencing. Substituting the structurally related DUF3715 domain of TASOR with either human or zebrafish TEX15 rescued HUSH activity in TASOR-deficient mESCs (109), again emphasizing the similarities between these RNA-directed silencing pathways.

HUSH therefore shares common features with both the RITS and piRNA silencing pathways, particularly in binding nascent RNA transcripts and using these transcripts to direct HUSH to the appropriate locus. HUSH also has unique features, having evolved a mechanism to recognize and silence its targets without the apparent need for small RNA intermediates or target amplification. The gain here is a silencing pathway that takes advantage of the unique features of single-exon, usually retroelement-derived, DNA to distinguish self from nonself and rapidly silence new RNA-derived elements following genome invasion. HUSH is therefore unique among other repressor complexes in that its silencing is innate and not learned or adaptive. This allows HUSH to play an essential role in genome immunosurveillance and provide front line defense by repressing the invading retroelement and inhibiting its transcription.

## Noncanonical HUSH Silencing Via NP220

Silencing by HUSH is not restricted to integrated transgenes, as HUSH also recognizes and silences a wide variety of unintegrated DNAs, which include unintegrated murine [Moloney murine leukemia virus (MLV)] (110, 111) but not human lentiviruses (112), as well as adeno-associated virus (113), adenoviruses (114), and herpes simplex virus (115). These studies identified NP220, a nuclear dsDNA-binding protein, as a new player in HUSH silencing (110). In a genome-wide CRISPR screen aimed to identify genes required for silencing nonintegrated MLV, similar to the approach used to study integrated retroviruses, NP220 and the core HUSH components and SETDB1 were identified as the main regulators of silencing (110). NP220 contains a DNA-binding domain and a C2H2-type zinc finger motif with a binding preference for cytidine clusters (116). Importantly, silencing of nonintegrated MLV was dependent on the recruitment of HUSH to DNA by NP220, as no HUSH components were recruited to DNA in the absence of NP220. Although NP220 could bind DNA without HUSH, this alone did not induce silencing (110). NP220-dependent silencing by HUSH therefore appears to be noncanonical, as it is dependent on NP220’s DNA-binding domain and would therefore appear to be RNA independent, though this needs to be formally demonstrated. NP220 recruits HUSH via MPP8, and as with canonical HUSH, silencing is dependent on SETDB1 and the deposition of H3K9me3, with an additional role for histone deacetylation via HDAC4 (110). A more extensive role for NP220 in HUSH silencing, beyond the silencing of unintegrated DNA, is anticipated and suggests HUSH is a versatile, modular silencing system that may use both RNA- and DNA-dependent mechanisms.

## Competition Between Two HUSH Complexes: The Role of HUSH2

The presence of a second HUSH complex had long been predicted, as a TASOR paralog (TASOR2) shares a similar domain organization and architecture to TASOR, with SPOC, DomI, and DomII/PIN domains. The TASOR2 protein is considerably larger than TASOR (2,645 amino acids versus 1,670), having an expanded disordered central region, and in its most common isoform, it lacks the N-terminal pseudo-PARP domain found in TASOR. AlphaFold structural analysis predicts TASOR2 to interact with MPP8 and periphilin and therefore form a repressor complex, potentially analogous to HUSH. However, TASOR2’s function remained elusive until two studies (117, 118) confirmed that TASOR2 does indeed associate with MPP8 and periphilin to form an independent complex, HUSH2, distinct from HUSH. TASOR and TASOR2 may therefore compete for binding to MPP8 and periphilin, though whether HUSH and HUSH2 are cooperative or antagonistic was unclear.

It was therefore essential to identify TASOR2/HUSH2-regulated genes. Rather than targeting retroelements such as L1s, RNA sequencing and genome occupancy analyses identified interferon-stimulated genes (ISGs) and KRAB-ZNFs as the two major target groups repressed by TASOR2 (117, 118). HUSH2 targets are not only distinct from HUSH but also show very different patterns of genome occupancy, with TASOR2-binding sites containing sharp, well-defined peaks distributed over gene promoters, coincident with active promoter marks. This contrasts with the broad distribution of TASOR over the body of HUSH target genes. How TASOR2 represses its targets remains unclear, as no H3K9me3 marks were detected at TASOR2/HUSH2 target sites. Importantly, TASOR2 is recruited to its ISG target genes by IRF2, a constitutively expressed ISG transcription factor (117, 118).

Despite the similarities between the HUSH and HUSH2 complexes, at first glance they appear to use different mechanisms to target unrelated loci. However, by competing for a limited pool of MPP8 and periphilin, the two HUSH complexes may coordinate their activities to enable immune activation in response to dynamic changes in the genome (117–119). For example, in response to increased retroelement activity, MPP8 and periphilin will be recruited away from HUSH2 targets to bind TASOR in attempts to control new HUSH-sensitive sites. If MPP8 and periphilin components are limiting, the HUSH2-targeted loci will be derepressed, resulting in increased ISG expression. Cooperation between HUSH and HUSH2 could therefore link the primary event, the HUSH-dependent recognition of nonself retroelements to two key downstream events: silencing of the retroelement by HUSH and subsequent immune activation through release of HUSH2-repressed ISGs. HUSH2 may also contribute to the retroelement-associated immune activation previously attributed solely to dsRNA detection (120). From a mechanistic viewpoint, recruitment of TASOR2 to its target ISGs by IRF2 resembles the NP220-mediated recruitment of HUSH to DNA discussed in the section titled Noncanonical HUSH Silencing via NP220 (110), as target recognition and subsequent HUSH2-dependent silencing is DNA-binding dependent and does not seem to require RNA binding. In further support of a non-RNA-dependent mechanism of HUSH2 recruitment, the marked enrichment of periphilin RNA transcript binding seen at TASOR/HUSH loci is not evident at TASOR2/HUSH2 loci (77). It will be interesting to determine whether the ZNFs regulated by TASOR2 also have immune activity and whether the components of the HUSH complexes are similarly regulated to maintain the balance between these two complexes.

## The Role of HUSH in Retroviral Silencing

The first description of TASOR restricting HIV infection predates the discovery of HUSH and emerged from a genome-wide siRNA screen that identified TASOR as a novel HIV-1 restriction factor (65). TASOR was subsequently shown to silence lentiviral reporters as well as HIV-1 provirus in models of latent HIV-1 infection (18, 71, 121). Complex primate lentiviruses encode a suite of viral accessory genes that antagonize host cellular restriction factors and therefore promote viral replication in cells that would otherwise restrict infection. While each viral accessory protein was initially thought to be responsible for the elimination of a single host cell restriction factor, unbiased approaches have shown how accessory proteins frequently antagonize multiple cellular targets (122, 123). Indeed, viral targeting of a host protein can highlight an unrecognized potential for resisting viral infection. The Vpr and Vpx proteins are unique among the lentiviral accessory proteins in being packaged into virions, allowing them to be immediately functional upon delivery to newly infected cells, without requiring de novo viral protein synthesis. The Vpr and Vpx proteins ubiquitinate and degrade TASOR by recruiting the host cell E3 ubiquitin ligase complex (DCAF1–CUL4), thus inhibiting HUSH activity (121, 123, 124). Reduced HUSH antiviral activity is therefore predicted to be required early in viral infection, prior to provirus integration. This Vpr/Vpx-mediated degradation of TASOR is seen across a wide range of diverse primate lentiviruses emphasizing the importance of HUSH in restricting these viruses. This function is more conserved than the previously described role for Vpx in degrading SAMHD1, a cellular phosphohydrolase that depletes cellular pools of dNTPs. Conservation of TASOR degradation by these viral proteins is all the more remarkable given the high variability and low amino acid identity (27%) of Vpx and Vpr sequences and may reflect the rapidly evolving host–pathogen interface (124). Vpx expression, as with HUSH depletion, accelerates the replication kinetics of primate lentiviruses (124), suggesting that HUSH represses primate lentivirus transcription and is antagonized by viral Vpx or Vpr proteins through HUSH degradation. The precise role of HUSH in silencing HIV-1, the most clinically important lentivirus, in particular the silencing of intact latent HIV provirus, requires further investigation, as HIV-1, unlike HIV-2, is one of the few primate lentiviruses whose accessory proteins do not degrade HUSH components. HIV-1 may have evolved alternative mechanisms of inhibiting HUSH. This area is of immense clinical relevance, as detailed evaluations of the HIV proviral integration sites in primary T cells from elite HIV controllers (125), patients who have received prolonged (>10 years) antiretroviral therapy (126, 127), and patients treated with latency-reversing agents (128) have all identified centromeric and peri-centromeric regions and the KRAB-ZNF gene locus on chromosome 19 as specific genomic hot spots where intact HIV-1 is maintained and persists (129), despite being under intense immune pressure to be eliminated. These sites are all notable for their sensitivity to HUSH-mediated silencing (18), making it important to determine the exact mechanisms facilitating viral persistence and the role of HUSH silencing at these loci.

## An Essential Role for HUSH in Early Development

The central role of HUSH in repressing RNA-derived elements, particularly L1 elements, suggests HUSH should be critical during early embryonic development. Indeed, all three core HUSH components are essential during development. The genome is particularly vulnerable to unregulated retrotransposition during the two waves of epigenetic reprogramming and global DNA demethylation that occur early in fertilization as the developing embryo transits through the zygote to blastocyst stages and during germline specification with the formation of nascent primordial germ cells (130–132). Periphilin or TASOR inactivation in mice causes peri-gastrulation lethality (at approximately embryonic day E7.5) with loss of cell fitness in TASOR knockout epiblasts and an increase in p53-driven apoptosis (64, 133, 134). Loss of MORC2A leads to embryonic lethality at approximately E13.5 (52) and loss of MPP8 is reported either to be lethal (before E11.5) (52) or result in offspring born at a decreased ratio with reduced body weight and size (135). Furthermore, specific deletion of either MPP8 or MORC2A in the murine nervous system increased expression of genes in the protocadherin gene cluster, affecting brain development and neuronal individuality (52).

All three HUSH components are also required for sustaining the self-renewal of pluripotent mESCs. While targeting MPP8 under serum/LIF culture conditions had minimal impact on cell growth, acute depletion of MPP8 in mESCs cultured in 2i/LIF (a hypomethylated state compared to serum/LIF) led to cell-cycle arrest in G1 phase and spontaneous differentiation. These findings correlate with their repression of L1 elements, suggesting that an intact HUSH complex helps repress retrotransposition at a critical stage when DNA methylation levels are low (53, 136).

HUSH’s role in the maintenance of naive pluripotency and in sustaining cell viability as cells transition from the naive state through to primed pluripotency was recently examined (137). The loss of TASOR led to replication stress, disrupted H3K9me3 heterochromatin, and sensitivity to increased L1 activity when exiting pluripotency. Whether the damage caused by this unregulated L1 activity is a consequence of genome damage from uncontrolled retrotransposition, toxicity of the L1 proteins, the generation of an innate immune response, or a combination of these effects was unclear. Partial protection of TASOR-knockout cells was afforded by either caspase inhibition or deletion of the mitochondrial antiviral signaling protein. This latter result suggests that dys-regulated L1 activity stimulates an innate immune response, consistent with reports that HUSH-dependent L1 upregulation stimulates a type I interferon (IFN) response initiated by MDA5 and RIG-I sensing of dsRNA release in human cell lines and primary human fibroblasts (120).

## Therapeutic Opportunities for HUSH Inhibition

Given HUSH’s role in repressing young L1 elements, HUSH inhibition may be of therapeutic benefit in three main areas: cancer immunotherapy, reversal of latent HIV-1 infection to achieve HIV cure, and improving gene expression. As HUSH depletion can induce IFN and an inflammatory response through L1 reactivation, targeting HUSH may increase tumor immunogenicity, with consequent IFN induction and increased antitumor immunity. Two studies have suggested a beneficial role for HUSH inhibition in cancers: Loss of function in vivo CRISPR–Cas9 genetic screens in mice identified SETDB1 and HUSH components as potential epigenetic checkpoints. Loss of these components sensitized solid tumors to immune checkpoint blockade, inducing the release of transposon-encoded neo-antigens and the generation of antitumor T cell responses (138). In contrast, an in vitro CRISPR–Cas9 genetic screen revealed MPP8-dependent L1 silencing as a selective dependency of acute myeloid leukemia (AML) cell lines (135). MPP8 inactivation was incompatible with the initiation or maintenance of in vivo models of AML but was not required for steady-state hematopoiesis. This was attributed to L1 reactivation inducing a DNA damage response, with inhibition of retrotransposition by reverse transcriptase inhibitors rescuing the genomic instability and growth defects of HUSH-deficient leukemic cells. These results implied a tumor-suppressive role for L1 retrotransposition in myeloid leukemia. Therefore, despite L1 elements being better recognized for their role in promoting cancer, HUSH inhibition and retrotransposition may also lead to tumor suppression, depending on the tissue and the tumor type. Tumors may be particularly sensitive to HUSH disruption, as in many cancers, the two-tier layer of epigenetic control is disrupted due to aberrant DNA methylation, leaving them more reliant on a functional HUSH for retroelement silencing.

HUSH is implicated in maintenance of latent HIV-1 provirus (see the section titled The Role of HUSH in Retroviral Silencing), and its inhibition may provide a means of reactivating latent provirus to help eliminate the persisting HIV-1 viral reservoir, the remaining obstacle to HIV-1 cure. This is the aim of shock-and-kill therapies, as reactivated provirus is readily killed by T cells in combination with antiretroviral therapy. The release of latent provirus reduces the latent viral load, with the aim of completely eliminating the proviral reservoir. HUSH inhibition is likely to play an important role, given the reports that remaining intact HIV-1 provirus is predominantly found in sites sensitive to HUSH silencing (129).

HUSH restricts expression of a large variety of integrated and nonintegrated cDNA transgenes, irrespective of their mode of delivery or whether they are integrating or nonintegrating. This includes lentiviral and adenoviral vectors, adeno-associated virus vectors, and nonviral vectors. Counteracting HUSH would therefore provide an effective means of improving gene expression with both in vitro and in vivo applications, such as increasing protein expression in the laboratory or enhancing expression with in vivo gene therapies. Modifications to improve lentiviral construct expression, e.g., the introduction of WPRE elements, have, albeit unwittingly, worked by counteracting HUSH repression. Further improvements that take into account HUSH’s mechanism of action will enhance transgene and vector design, e.g., insertion of intronic sequences or genetic/chemical HUSH inhibition to improve gene expression. As Cas9 transgenes are HUSH sensitive (29) and their expression is often rate-limiting, CRISPR–Cas9-related applications are also likely to benefit from HUSH inhibition.

## Conclusion and Perspectives

In the ten years since its original description, a combination of genetic, biochemical, evolutionary, and genomic analyses have shaped our current understanding of HUSH as an epigenetic transcriptional repressor complex, able to recognize and rapidly silence newly invading retroelements, and a new and unanticipated component of the innate immune system. HUSH discriminates self from nonself genomic DNA through the recognition of long, intronless DNA, the essential hallmark of reverse transcription. In so doing, HUSH provides a genome-wide immunosurveillance system, monitoring the genome for newly integrated retroelements and therefore defending the genome against the reverse flow of genetic information.

Perhaps the most important future goal is to gain further mechanistic insight into HUSH function in order to understand the nature of the signals that trigger HUSH binding to nascent transcripts and the subsequent events leading to assembly of the HUSH complex on chromatin. In particular, what features of transcription through long exons trigger HUSH binding and repression? Key to solving these problems are an understanding of the function of TASOR, which plays a central role in HUSH silencing but whose function remains enigmatic. These insights will be facilitated by structural information on HUSH components, the assembly of the HUSH complex as a whole, and the interactions of HUSH with RNA and chromatin. Equally important is furthering our understanding of the role of HUSH2 and how the dynamic interplay between the two HUSH complexes regulates the immune response following retroelement recognition. Although these goals are tractable, the dynamic nature of HUSH activity makes such analyses challenging, and structural studies have been hampered by the difficulties in expressing HUSH components in vitro. A further goal is to understand the attributes of introns that provide resistance to HUSH-mediated silencing and how this relates to their nucleotide composition, particularly the roles of A and T in conferring sensitivity or resistance to HUSH. Conversely, many endogenous intronless genes have learnt how to evade HUSH, as these genes are themselves retrocopies and therefore the product of retrotransposition events. Further examination of these genes should provide insights into the relationship between HUSH-dependent silencing and escape from HUSH via the RNA nuclear export pathways. The role of H3K9me3 in HUSH-mediated silencing, how H3K9me3 affects transcription over HUSH targets, and the function of H3K9me3-negative, HUSH-bound loci are also important to determine. Gaining further insight into HUSH’s role in vivo, the basis of HUSH’s requirement for embryonic development, and its role in germline specification will be of particular interest and importance. A more complete picture of HUSH function will provide further fundamental insights into self–nonself discrimination at the genomic level and help pave the way toward potential therapeutic benefits through harnessing HUSH activity.
